# Substituent size versus metal binding of inhibitors with variants of influenza endonuclease^☆^

**DOI:** 10.1016/j.jinorgbio.2025.113210

**Published:** 2025-12-31

**Authors:** Alysia J. Kohlbrand, Ryjul W. Stokes, Banumathi Sankaran, Seth M. Cohen

**Affiliations:** a Department of Chemistry and Biochemistry, University of California, La Jolla, CA 92093, USA; b The Berkeley Center for Structural Biology, Advanced Light Source, Lawrence Berkeley National Laboratory, Berkeley, CA 94720, USA

**Keywords:** Endonuclease, Influenza, Inhibitor, Mutants, Metal-binding pharmacophore

## Abstract

The influenza virus causes a significant burden of illness each year. Although vaccination is the most effective method to prevent seasonal influenza infection, viral escape mechanisms make vaccine composition difficult to predict. Antivirals are crucial for decreasing rates of morbidity and mortality from influenza viral infection. The newest anti-influenza drugs target the RNA-dependent RNA polymerase acidic N-terminal (PA_N_) endonuclease, a critical component of influenza viral replication machinery. This study examines the structure of inhibitors of PA_N_ that utilize a hydroxypyridinone-based metal-binding pharmacophore (MBP). Specifically, this report explores how the size of substituent groups impacts the binding conformation and affinity of a series of compounds against both wild-type (WT) and resistance mutant strains, I38T and E23K. Co-crystal structures revealed that the distance between compounds and enzyme residue 38 was conserved to maintain strong interactions, resulting in deviations from ideal coordination geometries at the active site metal centers. This suggests the interactions with residue 38 with each compound is important and can impact inhibitor potency as a consequence of distortions in the metal binding geometry of the compounds.

## 1. Introduction

Influenza is a viral disease of global importance with moderate morbidity and mortality, that involves epidemics and outbreaks alternating annually during the winter seasons of the northern and southern hemispheres [1]. In the past century, there have been five viral pandemics, four of which were due to influenza, with the most recent influenza pandemic being the 2009 “Swine Flu” H1N1 pandemic [2–4]. Influenza pandemics and epidemics have high attack rates, meaning a high percentage of the population is at risk of infection even with prophylactic measures such as annual vaccination [2]. Antivirals for influenza are a crucial second line of defense, especially during resurgence, epidemics, and pandemics. The anti-influenza drug Xofluza, also known as Baloxavir marboxil (**BXM**), was approved for therapeutic use and is the newest first-in-class drug for influenza in over 20 years [5]. **BXM** is a prodrug that is converted by hydrolysis to its active form, baloxavir acid (**BXA**).

**Fig. 1.**
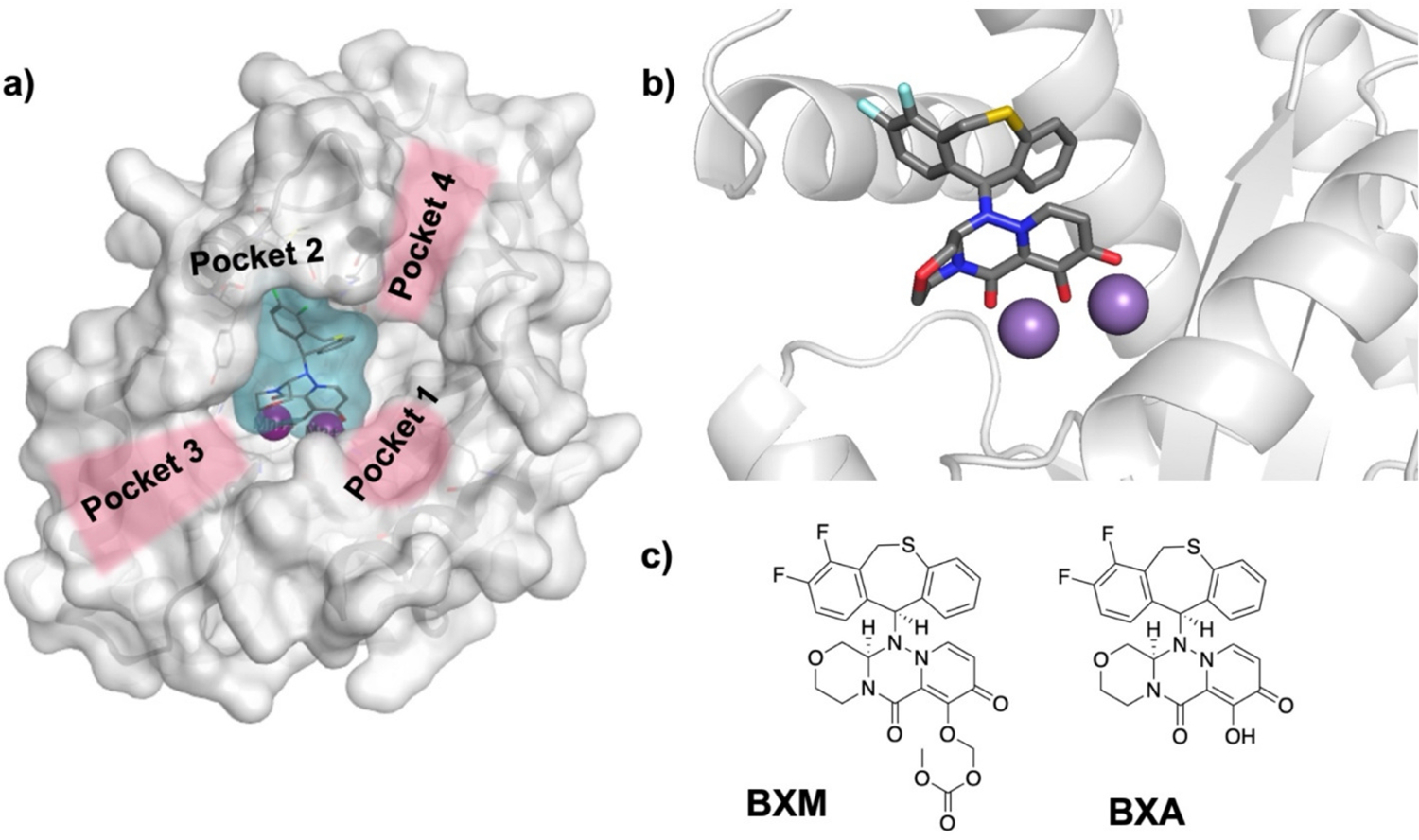
a) Surface representation of WT PA_N_ with baloxavir acid (**BXA**) bound, showing pockets 1–4. Pockets 1, 3, and 4 are visible in this orientation; pocket 2 is under the plane of the page. The **BXA** molecule is shown bound in pocket 2, with the Van der Waals surface representation shown in blue. b) Close-up view of BXA molecule bound to Mn^2+^ ions, with the I38T residue mutation highlighted in yellow. c) Structures of BXM and BXA. The protein backbone (gray) is shown as a cartoon, and Mn^2+^ ions are shown as purple spheres.

The target of **BXM** is the RNA-dependent RNA polymerase (RdRp) of the influenza virus, which aids in evasion of mammalian self-versus non-self RNA immune responses by stealing 5’-mRNA cap from host transcripts in a process referred to as “cap-snatching” [6]. The influenza virus RdRp complex is a heterotrimeric complex composed of PA, PB1, and PB2 subunits. The polymerase subunit PB2 captures host pre-mRNA, and PA_N_ cleaves 10–13 nucleotides to serve as a primer for viral mRNA synthesis by the PB1 subunit [7,8]. The PA_N_ active site is a large open cavity surrounding the divalent metal center with conserved distal patches within the cavity. This suggests that the RNA binding surface is extensive and there are a number of binding pockets amenable for inhibitor binding [8]. The exact mechanism for RNA cleavage is poorly understood, but thought to follow the PD-(D/E)XK nuclease superfamily [7]. Crystal structures of short oligomers and cryo-EM structures of the entire heterotrimer are available and provide additional insights into viable options for drug discovery [8,9]. Central to the active site, two Mn^2+^ cations are coordinated by His41, Glu80, Asp108, Glu 119, and Ile120 and a total of five water molecules, producing an octahedral coordination geometry at each metal center (Fig. 1). Surrounding the metal active site are four large hydrophobic pockets [10–12]. Pockets 2 and 4 are the main binding pockets for RNA binding and have been the most extensively explored for recent drug development (Fig. 1) [13–17]. Pockets 1 and 3 are more distal but are likely involved in shuttling RNA to and from other polymerase subunits (Fig. 1). This study focuses on pockets 2 and 4, thus purified PA_N_ endonuclease domain over the full heterotrimer was used for ease of expression, purification, and crystallization.

As a first-in-class inhibitor of PA_N_, **BXM** has been shown to reduce fever in an average of 24 h and is an oral monotherapy that can be taken at the time of diagnosis, which ensures higher patient compliance than medications taken over several days. Despite its clinical success, **BXM** has also demonstrated a high susceptibility to treatment-emergent resistance and viral rebound. In resistance monitoring studies following the approval of **BXM**, many treatment-emergent resistance mutations were identified [18,19]. The highest change in susceptibility and most prevalent resistance mutations found were in isoleucine 38 with I38T/M/F/L/N/S mutations. Other emergent mutations of interest are E23K and A36V mutations, which were also identified as resistance mutations resulting from **BXM** treatment [19]. **BXA** binds to the active site metal ions, while the “butterfly” motif found outside the heterocyclic, metal-coordinating core of the molecule provides extensive interactions with hydrophobic pockets 2 and 4 of the active site [19]. As **BXA** binds primarily to pockets 2 and 4, the resistance mutations identified are unsurprisingly also in pocket 2 (E23K) and pocket 4 (I38T and A36V). Finding other PA_N_ inhibitors with binding modes that occupy different active site pockets will be valuable for the treatment of circulating resistance mutations.

This study investigates a series of inhibitors based on a hydroxypyridinone (HOPO) metal-binding pharmacophore (MBP) appended with functionalized phenyl backbone groups (Fig. 1). Inhibitor affinity was determined by biochemical and biophysical methods against the WT enzyme and clinically important mutants, I38T and E23K. Using these methods, the impact of the substituents of the phenyl ring with changes in the protein construct was quantified. Co-crystal structures revealed that the distance between compounds and amino acid residue 38 was conserved to maintain an optimal contact. By maintaining this peripheral interaction with residue 38, deviations from ideal binding geometries at the metal active site by the inhibitors was observed. This suggests the Van der Waals interactions at residue 38 with each compound is quite strong, and the loss of activity of inhibitors is likely due to the resulting distortions in the metal binding geometry (Fig. 1).

## 2. Experimental

### 2.1. Mutation generation

Point mutations were generated in the PA_N_ endonuclease pET 28a plasmid by QuikChange mutagenesis (Agilent). Each PCR reaction of 50 μL contained 50 ng of template, 125 ng primer pair, 200 μM dNTPs and 3 units of Pfu DNA polymerase. The PCR cycles were initiated at 95 °C for 1 min to denature the template DNA, followed by 12 amplification cycles. Each amplification cycle consisted of 95 °C for 50 s, 60 °C for 1 min, and 68 °C for 6 min. The PCR cycles were finished an extension step at 68 °C for 7 min. The PCR products were treated with 5 units of *Dpn*I at 37 °C for 1 h and transformed into ultra competent cells and plasmid extracted. All mutations were first verified by Sanger Sequencing (Eton Biosciences) and then full, Non-Sanger plasmid sequencing (Primordium Labs) to ensure no other mutations occurred during the QuikChange prior to expression and purification (Table 1).

### 2.2. Protein expression and purification

Expression and purification of PA_N_ endonuclease was performed with slight modification of a reported procedure [20]. The pandemic isolate A/California/04/2009(H1N1) N-terminal PA (PA_N_) endonuclease Δ52–74:Gly truncated construct was expressed from a pET-28a parent vector containing a kanamycin-resistance reporter gene with expression inducible by the Lac1 operon. PA_N_ endonuclease was expressed as an 8-histidine tagged fusion protein cleavable by TEV protease. The transformation protocol was adapted from xpET system manual (Novagen) using single competent BL21 cells. Briefly, 1 μL of 25 ng/μL recombinant plasmid was used for transformation. Cells were mixed with the plasmid and were heat shocked at 42 °C for 30 s followed by incubation on ice for 3 min. Outgrowth was plated on LB agarose plates containing 50 μg/mL kanamycin and was incubated overnight at 37 °C. One colony was scraped from the LB plate and added to 5 mL of SOC broth containing 50 μg/mL kanamycin and was incubated overnight at 37 °C with shaking at 125 rpm. SOC media (100 mL) containing 50 μg/mL kanamycin was combined with the 5 mL overnight growth and was incubated with shaking at 200 rpm at 37 °C until the OD_600_ of this starter culture reached >2 (3–4 h). The culture was equally divided into 6, 2 L flasks containing 1 L of expression media (TB media with added 0.2 % dextrose, 0.1 mM MnCl_2_, and 0.1 mM MgSO_4_, 50 μg/mL kanamycin). Cells were grown to and OD_600_ between 0.4 and 0.6 at room temperature with shaking at 200 rpm (3–4 h). Expression was then induced by addition of IPTG to a final concentration of 0.1 mM. The cultures were grown with vigorous shaking (250 rpm) overnight at room temperature. After ~18 h the cells were harvested by centrifuging at 2000 g for 30 min at 4 °C. The resulting paste was stored at −80 °C prior to lysis.

Cell paste was thawed in batches on ice for 2 h and resuspended in 25–35 mL of lysis buffer (1 % Triton-X, 1 mM MgCl_2_·6H_2_O, 2 mM DTT, 10–100 μg/mL DNAse-1, 1 mg/ml lysozyme, and 1 % glycerol) and EDTA free protease inhibitor (Roche). Cells were lysed using a probe sonicator (Fisherbrand model 120) with cycles of 25 s pulses and 59 s rest at 60 % amplitude. Cell debris was then pelleted by centrifugation at 10000 rpm for 45 min at 4 °C. The supernatant was decanted from the pellet, and a HisTrap FF (Cytiva) column was utilized to isolate Histagged fusion protein from the cell lysates according to the manufacturer’s recommendations at 4 °C. Briefly, cell-free lysates from 6 L growth were loaded on 5 mL column that had previously been charged with Ni ions. The column was then washed with binding buffer (20 mM Na_2_PO_4_, 500 mM NaCl, 25 mM imidazole, pH 7.4) until fraction absorbance reached a steady baseline. The protein was then eluted over a gradient from 0 to 100 % elution buffer (20 mM Na_2_PO_4_, 500 mM NaCl, 500 mM imidazole, pH 7.4) at a flow rate of 4 mL/min. PA_N_ endonuclease eluted between 40 and 60 % elution buffer. SDS-PAGE analysis showed a band corresponding to PA_N_ endonuclease running at ~23 kDa with several small impurities.

Fractions containing PA_N_ endonuclease were combined in a 10 K MWCO dialysis bag with 1000 units of TEV protease and were dialyzed against dialysis buffer (100 mM NaCl, 1 mM dithiothreitol, 1 mM MnCl_2_, 20 mM Tris, 5 % glycerol, pH 8.0) overnight with three buffer exchanges. The proteolytic cleavage of the fusion protein is slow and greatly benefits from the addition of excess TEV protease. A white precipitate forms over time. After buffer exchange, the solution was filtered through a 0.45 μm filter. The solution was run through the Histrap FF column equilibrated with the same binding buffer as before. The resulting flow through contained His-cleaved PA_N_ endonuclease, which was then concentrated to 5–10 mg/mL using a pressurized Amicon and/or spin Amicon concentrator. The concentrated protein was then purified on a gel-permeation size exclusion column (GE Superdex 75, 10/300 GL) according to manufacturer recommendations in buffer (150 mM NaCl, 2 mM MgCl_2_, 2 mM MnCl_2_, 20 mM HEPES, pH 7.5). A large peak corresponding to the cleaved PA_N_ endonuclease eluted at ~12 mL eluent. A small shoulder before the main peak was occasionally observed, which contained primarily uncleaved and/or unfolded PA_N_ endonuclease construct. Fractions containing pure cleaved PA_N_ endonuclease were combined and concentrated to 2–5 mg/mL. Stored protein was flash-frozen in liquid nitrogen and was kept at −80 °C. This protein was suitable for use in enzyme or thermal shift assays or for protein crystallography.

### 2.3. Differential scanning fluorometry (DSF)

DSF experiments were performed according to a reported procedure [20,21]. The apparent dissociation constant (*K*_d_) was calculated by comparing the change in melting temperature vs. ligand concentration using GraphPad Prism to determine apparent ligand affinity (*K*_d_) [21,22]. Curve fitting was based on the equation:

$$\text{Y}=\text{Bottom}+\left(\left(\text{Top}-\text{Bottom}\right)*\left(1-\left(\text{P}-K_{d}-\text{X}+\sqrt{\frac{\left(P+X+K_{d}^{2}\right)-\left(4PX\right)}{2P}}\right)\right)\right)$$

where Bottom = native melting temperature, Top = max temperature, X = ligand concentration in uM, *K*_d_ = same log unit as X, and P = protein concentration. Rules for initial values in GraphPad Prism were: Top = Y_MAX_, Bottom = Y_MIN_, *K*_d_ = value of X at Y_MID_, and P = constant equal to [21,22]. Concentrations used were 0, 25, 50, 100, 200 and 1000 μM. Compound **4** was poorly soluble at 1000 μM so only 0–200 μM points were used. All assay conditions apart from ligand concentration, such as protein and DMSO concentrations, were held constant. Each well of a 96-well 0.2 mL optical MicroAmp (ThermoFisher) thermocycler plate contained a volume of 20 μL containing final concentrations of 1 μg PA_N_ endonuclease, 25–1000 μM inhibitor, and 1 × SYPRO orange Thermal Shift dye in buffer (150 mM NaCl, 2 mM MnCl_2_, 20 mM HEPES pH 7.5) with 4 % DMSO. A master mix containing PA_N_ endonuclease, SYPRO orange Thermal Shift dye, and buffer was made and 16 μL added to each well. Either 1 mM inhibitor stocks or 20 % DMSO stocks were made, to which 4 μL was added to the plate to make a total of 20 μL final volume. Each well was mixed thoroughly by pipetting up and down, with care to prevent air bubbles from forming. The presence of this small concentration of DMSO was found to have a negligible effect on ΔTM values of native PA_N_ endonuclease. Thermocycler plate wells were sealed prior to analysis, and the plate was then heated in a thermocycler from 25 to 99 °C at a ramp rate of 0.05 °C/s. Fluorescence was read using the ROX filter channel (λ_ex_ = 580 nm; λ_em_ = 623 nm), and the fluorescence signal was fitted to a first derivative curve to identify TM. All assayed compounds were observed to cause either no significant Δ*T*_M_ or a positive Δ*T*_M_.

### 2.4. Protein crystallography

Crystallization of PA_N_ endonuclease was performed with slight modification of a reported procedure [23]. Purified protein for crystallization was stored at 2.2–4.3 mg/mL at 80 °C after flash freezing in buffer consisting of 150 mM sodium chloride, 20 mM HEPES (pH 7.5), 2 mM MgCl_2_, and 2 mM MnCl_2_. Co-crystallization and crystal soaking methods were used to obtain co-crystal structures of inhibitors bound to PA_N_ endonuclease. BXA was purchased from Fisher and used without further purification. Compound 23 was previously synthesized according to Credille, et. all [20]. For co-crystallization, protein was incubated with 0.5 mM inhibitor for 1 h on ice prior to setting the crystallization drops. For crystal soaking, fully formed holo crystals were transferred to a new drop containing 5 μL of reservoir solution and 1 μL of 50 mM DMSO inhibitor stock solution (final concentration 8.3 mM). Crystals were left undisturbed overnight and either stored in liquid nitrogen or collected on an in-house X-ray diffractometer the following day. In both crystallization methods, crystals were grown using hanging drop and set in 24-well pre-greased plates (Hampton HR3–171) with siliconized glass slides (Hampton HR3–231). A 5:1 ratio of purified protein to reservoir solution at room temperature was found to be the optimal ratio for the largest crystal formation. Reservoir solution consisted of 22–34 % PEG (MW 4000 g/mol), 100 mM Tris (pH 8.35), and 220 mM sodium acetate. Colorless crystals with hexagonal bipyramidal morphology appeared within 2 days and reached full size after 1–2 weeks. Crystals were typically 50 to 200 μm in diameter. Crystals were cryoprotected with perfluoroether (Hampton HR2–814) prior to flash freezing in liquid nitrogen. Crystals were stored in liquid nitrogen until data collection.

Datasets for the following structures, I38T with compound **3** (PDB: 9PN2), E23K with compound **4** (PDB: 9PNL), and WT with compound **4** (PDB: 9PO6) were collected at the Advanced Light Source (ALS), Lawrence Berkeley National Laboratory, in collaboration with Dr. Banu Sankaran through Collaborative Crystallography program. The datasets for I38T with compound **1** (PDB: 9PMP), I38T with compound **2** (PDB: 9PMR), I38T with compound **4** (PDB: 9PN3), and WT with compound **3** (PDB: 9PNM) were collected at the Stanford Synchrotron Radiation Lightsource (SSRL), Stanford Linear Accelerator Center (SLAC) National Laboratory on beamline 12–1. For all datasets (including datasets collected on the in-house X-ray generator), phasing was determined by molecular replacement against a previously published PA_N_ endonuclease structure (PDB 8DDB) using PHASER. All structures were refined with Phenix version 1.19.2. Crystal and refinement statistics are provided in Table 2.

## 3. Results and discussion

### 3.1. Biochemical and biophysical evaluation

**Fig. 2.**
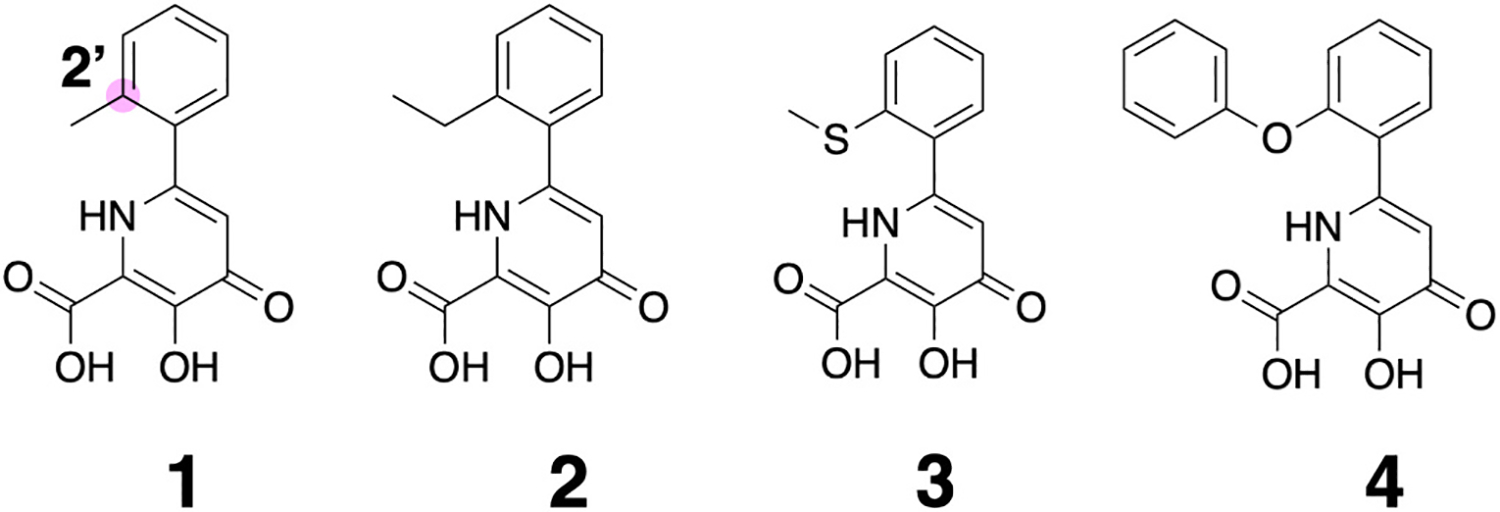
Chemical structures of compounds investigated in this study. The 2′-position in compound **1** is labeled for clarity.

A prior screen of the inhibitors shown in Fig. 2 showed that substituents at the 2′-position revealed that small substituents such as methyl, ethyl, and trifluoromethyl produced the most active compounds [20]. Adding small alkyl substituents at the 2′-position was determined to enhance favorable hydrophobic interactions with in the active site walls. It was also hypothesized that the trifluoromethyl and methyl substituents in the 2′-position provided an optimal binding distance to the active site wall and allowed the inhibitors to maintain idealized octahedral metal coordination geometry. Mutation of Ile38 to Thr38 results in removal of a methyl group on the active site wall. It was hypothesized that compound **2** with an ethyl substituent at 2′-position (Fig. 2) would have similar inhibitory activity and thermal melting stability when compared to compound **1** (with a methyl in the 2′-position) against the WT and I38T mutant enzymes.

Due to the high affinity of our inhibitors, determining the inhibitory activity by measuring IC_50_ values using conventional FRET-based enzymatic assays is not particularly informative, as many inhibitors will display activity at concentrations below the sensitivity threshold of the assay [20,23,24]. For example, the activity of compounds **1** and **2** against WT enzyme using FRET-based assays showed comparable activity with pIC_50_ values of <2.0 nM [20], and compounds **3** and **4** were on the edge of the limit of detection of the FRET-based assay, with IC_50_ values reported to be ~3 nM. Similar limit of detection issues were also a challenge in a prior study of **BXA** [23].

**Fig. 3.**
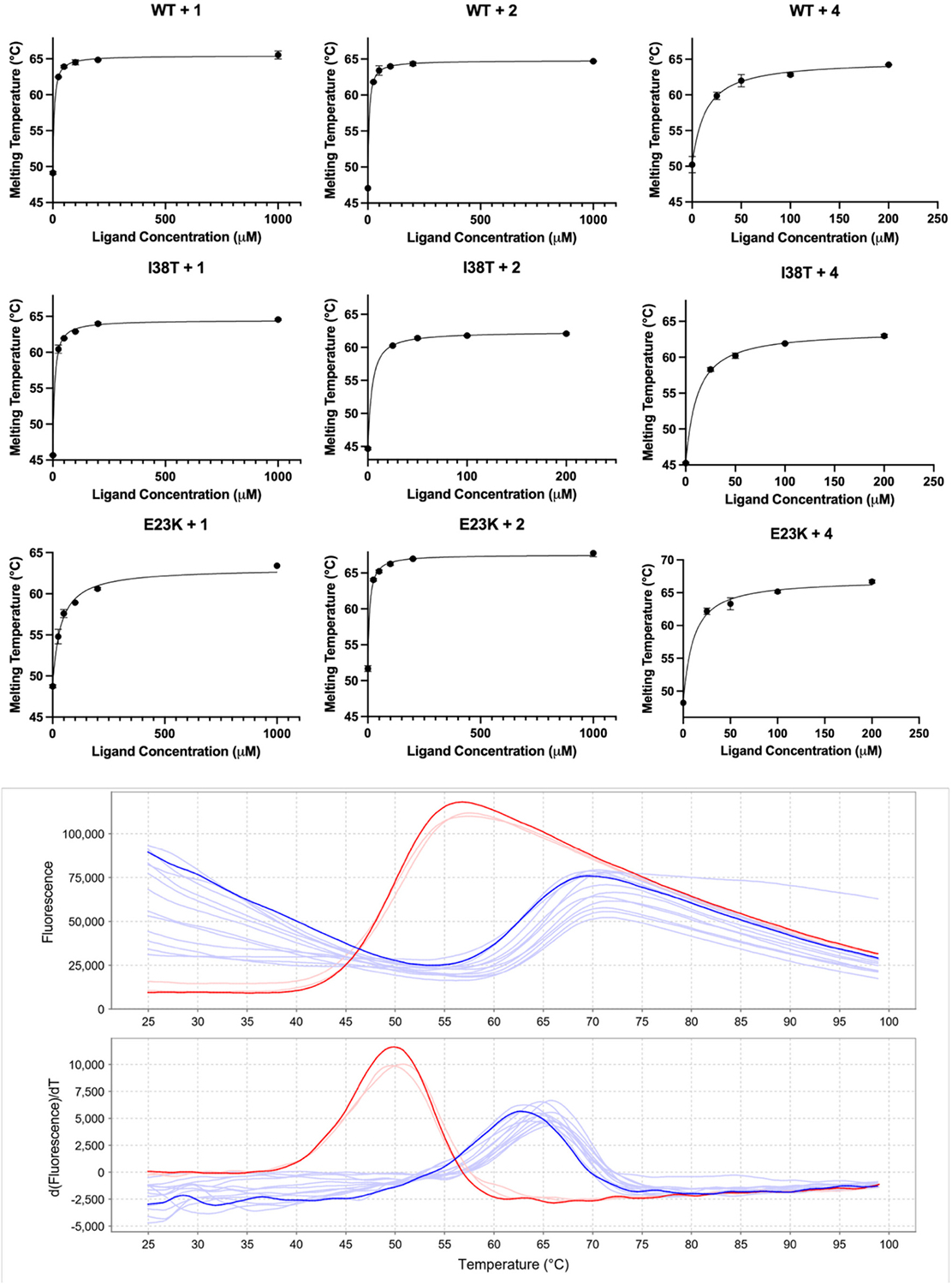
*Top*: Apparent *K*_d_ curves for compounds **1**, **2**, and **4** against WT, I38T, and E23K. The concentration range is 25 μM to 1 mM. Values and 95% confidence interval (CI) are given in Table 4. Graphs were generated using GraphPad Prism. *Bottom*: Fluorescent melting curve trace and first derivative of the fluorescent trace. The maximum of the derivative peak for each trace is the melting point of the protein. Representative data is shown as WT melting curve in the presence (blue) and absence (red) of compound **1**.

The thermal stability of a protein generally increases upon formation of favorable protein–ligand contacts, with tighter-binding interactions typically resulting in larger changes in thermal shift (Δ*T*_M_) compared to an apo (ligand-free) control. In previous studies, differential scanning fluorimetry (DSF) assays or spectral shift (SpS) methods were used to identify the change in Δ*T*_M_ between inhibitor-free and inhibitor-bound PA_N_, and a strong correlation was observed between inhibitory activity and Δ*T*_M_ across multiple inhibitor chemotypes [20]. Using this relationship, the pIC_50_ values of compounds with common chemical features can be extrapolated to estimate values that are otherwise below the detection limit of the FRET-based nuclease activity assay [25]. Compounds **1**–**4** were evaluated in a thermal shift assay to approximate the dissociation constant and calculate apparent *K*_d_, and compare single-point measurements against WT PA_N_ endonuclease and I38T and E23K mutants (Table 4 and Fig. 3). Compound **1** shows an apparent *K*_d_ value of 6.84 μM and Δ*T*_M_ value of 9.18 °C at 200 μM with the I38T mutant, which suggests weaker binding when compared to WT (5.18 μM and 11.91 °C). Affinity is recovered with compound **2**, where an apparent *K*_d_ of 3.06 μM and a thermal shift value of 11.16 °C at 200 μM (vs. 4.57 μM and 11.82 °C with WT enzyme) is observed. No other significant changes in Δ*T*_M_ were observed with the remaining compounds, indicating that binding of compounds **1**–**4** are not largely affected by the I38T mutation (Table 3, Table 4, and Fig. 3). The E23K mutation has been found to generally produce weaker apparent *K*_d_ values with inhibitors due to changes in the stability of the construct. That same observation can be made here with typically larger apparent *K*_d_ values and smaller Δ*T*_M_ values at 200 μM (hence weaker binding, Tables 3 and 4) of compounds **1**–**4** with the E23K mutant when compared to the WT enzyme. There is one interesting outlier in compound **4**, where binding to the E23K mutant is better (9.31 μM and 9.91 °C) when compared to WT PA_N_ (12.34 μM and 6.46 °C). This suggests the formation of a new interaction between the E23K variant and compound **4**. These changes are interesting questions that lend themselves to structural characterization, and as such, compounds **1–4** were selected for co-crystallization studies.

### 3.2. Structural studies of compounds 1–4 with PA_N_ endonuclease variants

**Fig. 4.**
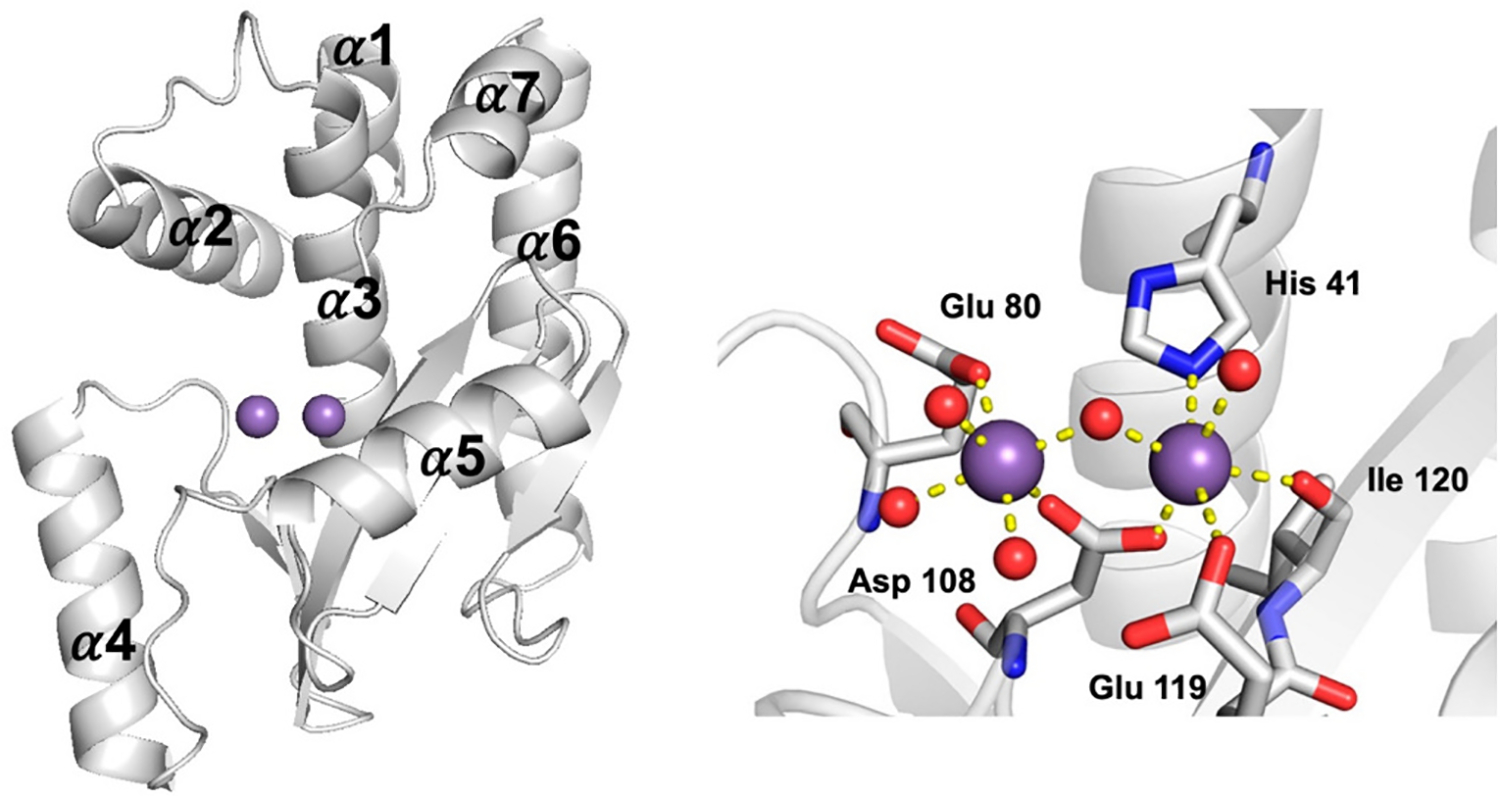
*Left:* WT holo structure with all alpha helices (*α*1–*α*7) labeled. *Right*: Close-up of holo Mn^2+^ ions with coordinating residues (His41, Glu80, Asp108, Glu119, and Ile120) and water molecules shown. The protein backbone (gray) is shown as a cartoon. Mn^2+^ ions and water molecules are shown as purple and red spheres, respectively.

PA_N_ is made up of seven core alpha helices (*α*1–*α*7), five beta sheets (β1–β5), and two Mn^2+^ ions responsible for catalysis (Fig. 4). The two Mn^2+^ cations are coordinated by His41, Glu80, Asp108, Glu119, and Ile120 and a total of five water molecules, producing an octahedral coordination geometry at each metal center [20,23,26] (Fig. 4) sur-rounded by four large hydrophobic pockets (Fig. 1) [10–12]. Pockets 2 and 4 are the main binding pockets for RNA binding and have been the most extensively explored for drug development. Pockets 1 and 3 are more distal but are likely involved in shuttling RNA to and from other subunits of the polymerase.

As a baseline, the inhibitor-free, holo structures of the I38T and E23K mutants were determined [23]. The I38T mutation is located in *α*3, and E23K is located in *α*2. No major structural changes were observed in the I38T holo structure compared to holo WT. [23] When comparing the holo structure of E23K with WT, the terminus of *α*2, where the E23K mutation is located, is distorted [23]. In WT PA_N_, Glu23 forms hydrogen bonds with Arg84, stabilizing the terminus of *a*2, which promotes the correct position of Tyr24 for base stacking. This interaction is not possible in the E23K variant, causing disorder of *a*2 [23].

**Fig. 5.**
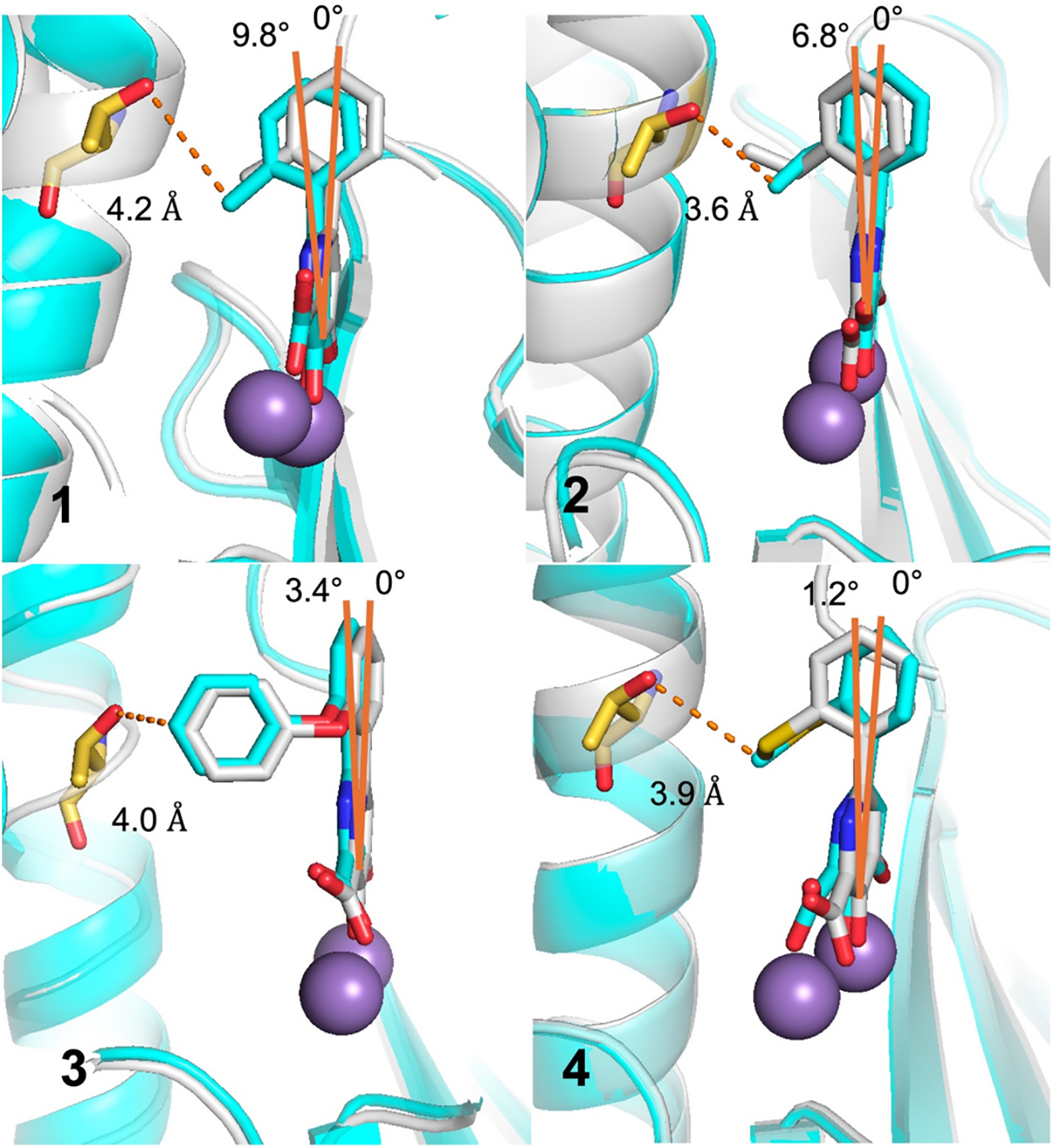
Distance to Ile38 (yellow) and binding angle comparison of compounds **1–4** bound to WT (gray) and I38T (cyan) variants of PA_N_. Binding is similar across variants with slight changes in binding angle and distance. The protein backbone (gray, cyan) is shown as a cartoon, and Mn^2+^ ions are shown as purple spheres.

As expected and described below, all the compounds studied here coordinate to the active site metal ions through a triad of oxygen donor atoms from the HOPO MBP, which include oxygen donor atoms from the ketone and carboxylic acid groups with a bridging phenolate [20,23,24,26,27]. The structure of compounds **1**–**4** were co-crystallized with WT PA_N_, as well as the I38T and E23K mutants of PA_N_. The results of these co-crystal structures are described below and descriptions of how these structures explain some of the trends observed in the biochemical and biophysical data are provided. Three important structural parameters are described as part of this analysis. First, the distance to the active site wall (*α*3 helix) and the terminal carbon of the 2′ substituents of the phenyl ring was calculated from the hydroxyl group of Thr38, or carbon 4 of Ile38 for all compounds and constructs (Fig. 5). Second, a ‘binding angle’ was calculated by comparing the vector of the MBP to the 4′ position of the phenyl ring of a compound in a mutant structure versus the same compound bound to the WT enzyme (Fig. 5). Third, the coordination bond distance between the MBP and the Mn^2+^ ions was examined. The average distance between the MBP donor atoms and the Mn^2+^ ions in this study (as well as previous reports) is ~2.2 Å, and significant variations (0.1 Å or greater) have been shown to lead to a distortion from idealized octahedral geometry [20,23,24].

**Fig. 6.**
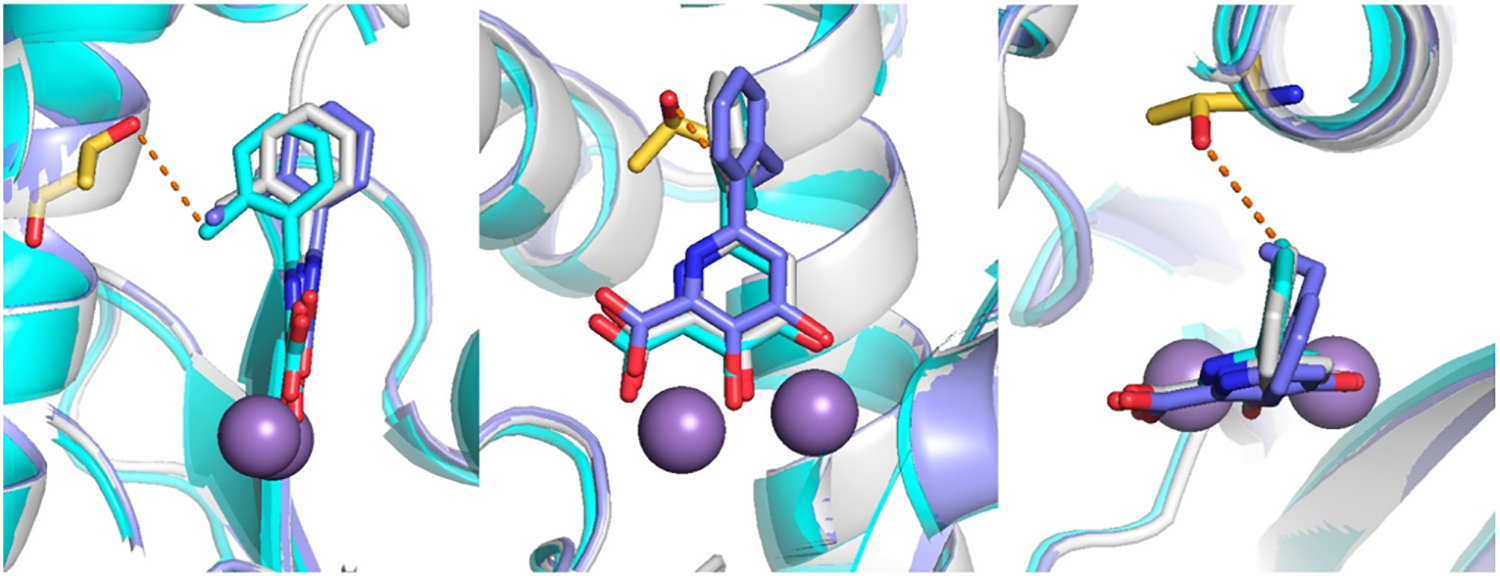
Three different views of the superposition of compounds **1** and **2** co-crystal structures with WT and I38T PA_N_. Compound **1** bound to WT is shown with the protein backbone and carbon atoms in gray, or bound to the I38T mutant in cyan. Compound **2**, bound to WT PA_N,_ is shown in with the protein backbone and carbon atoms in purple. The key observation from these superposition images is that the contacts between the inhibitors and residue 38 are maintained despite changes in the protein backbone and substituent. The protein backbone is shown as a ribbon (gray, cyan, purple), and Mn^2+^ ions are shown as purple spheres.

Compound **1** possesses the smallest 2′ substituent among the HOPO derivatives, namely a methyl group (Fig. 5). This methyl group makes favorable Van der Waals interactions with Ile38 of WT PA_N_. Extending the methyl group to an ethyl group in compound **2** when bound to the WT enzyme causes a slight tilt outward (relative to the WT structure) of 7.2 so that the terminal carbon of the ethyl group at 2′-position makes similar contacts as the methyl group in compound **1** (Fig. 4) where the same distance of ~3.7 Å to Ile38 is maintained. Comparing the WT and I38T structures of compound **1**, a similar trend in binding angle is observed where compound **1** tilts 9.8° inwards (relative to the WT structure) towards Thr38 in the I38T structure, maintaining the same 3.7 Å distance seen in WT structures of compounds **1** and **2** (Figs. 5 and 6). Interestingly, the activity of compound **2** against I38T is comparable to that of compound **1** against WT. Unsurprisingly, trends in binding angle and inhibitory activity are manifest as changes in the binding geometry of the compounds to the metal centers. The MBP-metal bond distances for compound **1** in the WT structure are ~2.2 Å, which has been shown in previous studies to be as close to ideal octahedral geometry as these ligands can achieve [23]. The MBP-metal bonds in compound **2** in the WT structure arê0.4 Å longer, suggesting a larger deviation from ideal octahedral geometry, and as such compound **2** is a weaker inhibitor (Fig. 5). Compound **2** in the I38T mutant structure is very similar to the structure with the WT protein, but with bond distances only ~0.1 Å longer than the compound **1** with the WT enzyme (Fig. 5). This correlates with a recovery of inhibition activity.

**Fig. 7.**
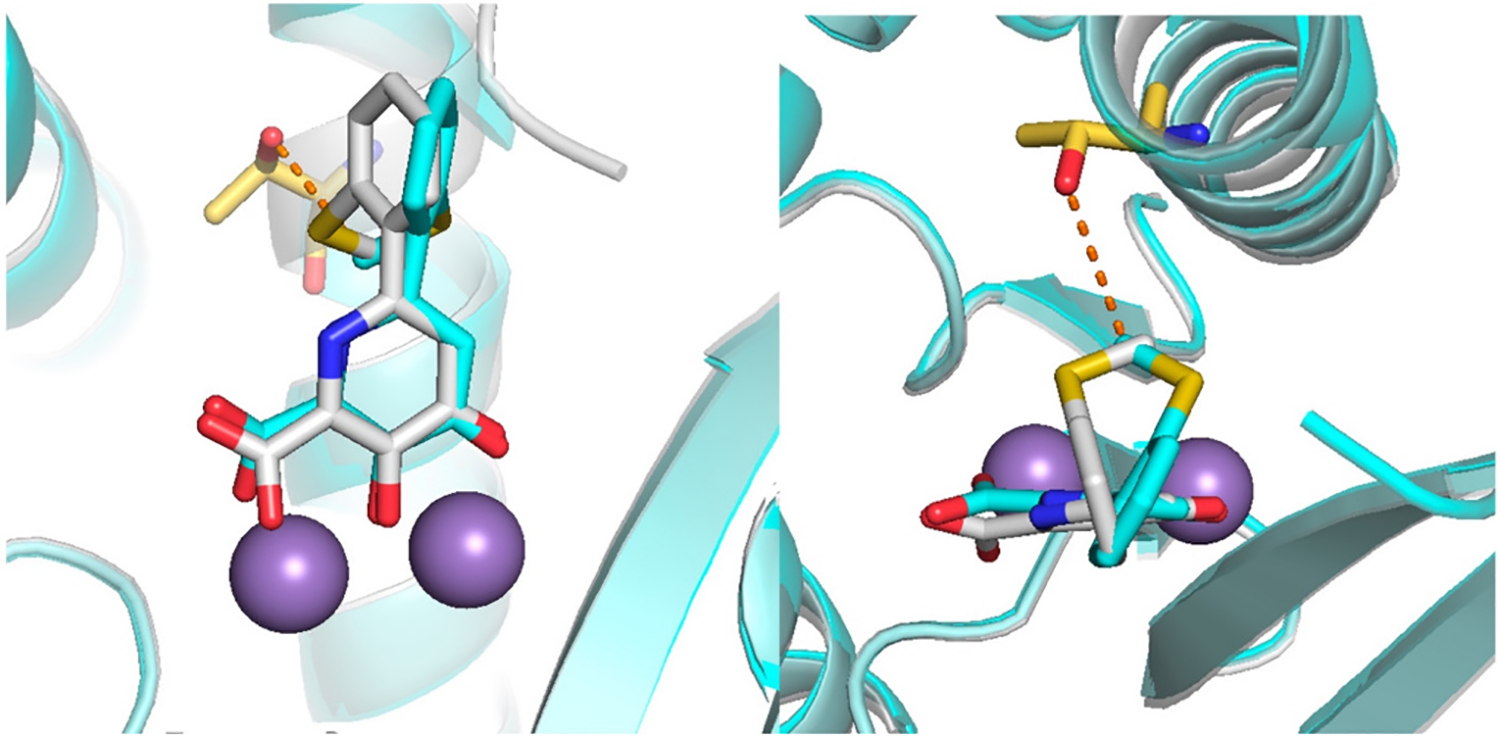
Binding comparison of compound **3** bound to WT (gray) and I38T (cyan, mutation in yellow) variants of PA_N_. Binding is similar across variants with slight changes in binding angle and thioether rotation. The protein backbone (gray, cyan) is shown as a cartoon, and Mn^2+^ ions are shown as purple spheres.

The thioether substituent at the 2′-postion of compound **3** is slightly larger than the ethyl substituent of compound **2**. No significant changes in binding angle or coordination geometry were observed when comparing the WT to the I38T structure, and the distance to residue 38 is maintained at 3.9 Å for both variants (Fig. 7). It appears there is more flexibility in substituent and phenyl ring rotation, as there is a perfect 180-degree rotation between the phenyl ring and thioether substituent between the WT and I38T structure (Fig. 7). This rotation could explain the slight decrease in activity and thermal stability measure for compound **3** against WT and the I38T mutant. The overall distance to Ile38 for compound **3** is longer than for compounds **1** and **2**, fitting the trend that increasing 2′ substituent size increases the outward tilt of the compound, decreases the inhibitory activity, and reduces thermal stability. The metal coordination bond length is also 2.4 Å, 0.2 Å longer than smaller substituents such as the WT compound **1** structure. This is also in agreement with the previous observations for compounds 1 and 2, comparing the WT and mutant structures.

**Fig. 8.**
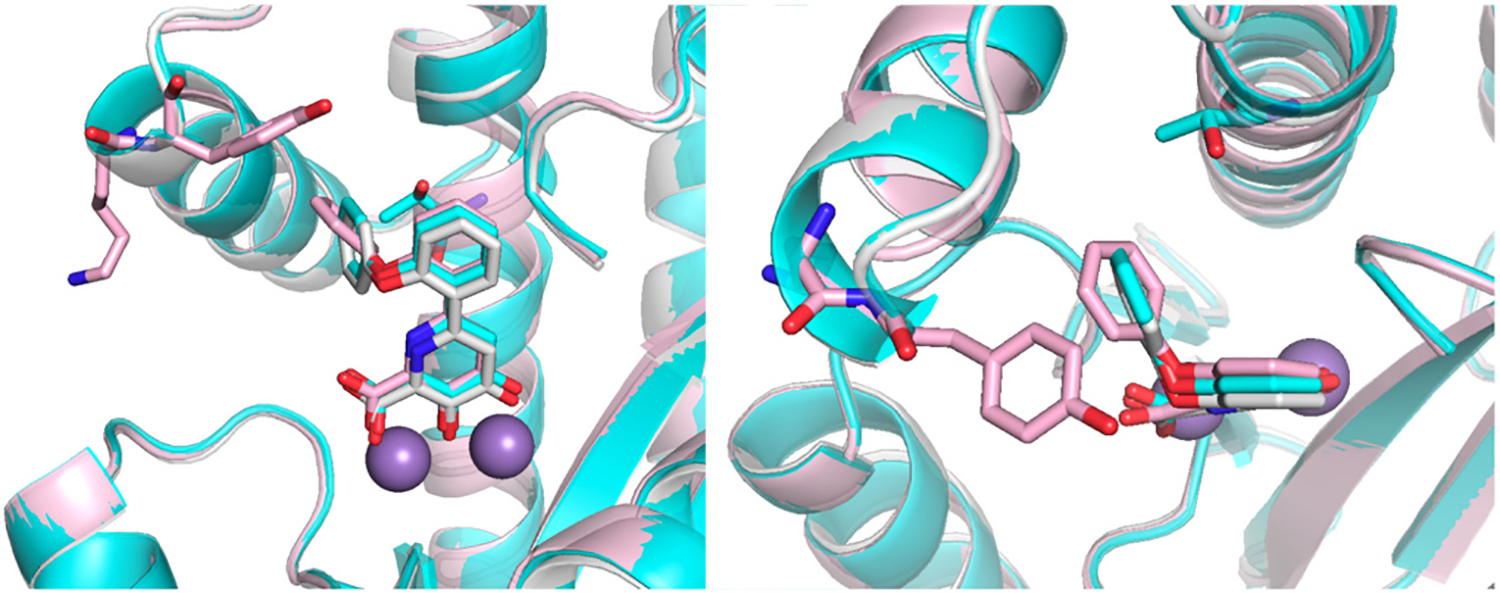
Binding comparison of compound **4** bound to WT (gray), I38T (cyan), and E23K (pink) variants of PA_N_. Binding is similar across variants with only slight changes in binding angle and phenolic ether rotation. The protein backbone (gray, cyan, pink) is shown as a ribbon, and Mn^2+^ ions are shown as purple spheres.

Larger 2′ substituents would logically require an even greater outward tilt to maintain the same van der Waals contact with residue 38, would need to face outward towards the solvent-exposed section of pocket 3, or would bind in pocket 2, where RNA bases stack during endolytic cleavage. Compound **4** has the largest 2′ substituent (phenolic ether) and binds in pocket 2 (Fig. 8). Surprisingly, compound **4** showed better inhibitory activity against the E23K mutation than the WT or I38T mutation. Comparing the three structures, compound **4** binds very similarly across each variant regarding binding angle and phenyl ring placement, but the phenolic ether of compound **4** is rotated in the E23K mutation compared to the WT and I38T (Fig. 8). The WT and I38T comparison of compound **4** is unsurprising, and the compound binds very similarly, but in the I38T mutant compound **4** has a slight inward tilt of 1.7° compared to WT. It is also unsurprising that the inward tilt is smaller than observed for compounds **1** (9.8°) and **2** (7.2) due to weaker Van der Waals forces with Ile38 or Thr38. This is observed in the change in the distance from position 38, going from 3.7 Å to 4.1 Å. The phenolic ester of compound **4** in the E23K mutation is rotated approximately 40° degrees compared to the other structures (Fig. 8). Surprisingly, Tyr24 does not pi-stack with compound **4** in the structure. However, the collapse of the terminus of *α*2 is not observed, like in the apo or structures with other compounds in this study or in previous studies [23]. Compound **4** may prevent Tyr24 from extending into the active site and restrict its freedom of movement. This could explain the restoration of inhibitory activity and Δ*T*_M_ (Tables 3 and 4). The metal coordination bond length is also 2.4 Å, as seen in structures of compound **3**.

## 4. Conclusion

This work describes the binding trends and structures of a series of HOPO-based inhibitors of PA_N_ with WT enzyme and two resistance-relevant mutants. The trend of increasing substituent size resulting in decreased inhibitory activity and thermal stability was linked to an increase in the outward tilt of the compound that distorts the optimal octahedral coordination to the Mn^2+^ active site ions. The recovery of inhibitory activity and thermal stability of compound **4** with the E23K mutation suggests that compound **4** prevents collapse of the terminus of *a*2 by preventing Tyr24 from extending into the active site and restricting its freedom of movement. The findings here suggest changing substituent size can modulate activity against the I38T and E23K mutations; however, additional studies should be performed to find substituents that are effective at restoring activity against both mutations for more mutation-resistant inhibitors.

## Figures and Tables

**Table 1:** QuikChange primers used in mutation generation.

Construct	Primer
I38T	F: CAAATTTGCAGCCACTTGCACCCATCTGGAAG
	R: CTTCCAGATGGGTGCAAGTGGCTGCAAATTTG
E23K	F: GCAGAGAAAGCCATGAAAAAATATGGTGAAGATCCG
	R: CGGATCTTCACCATATTTTTTCATGGCTTTCTCTGC

**Table 2:** X-ray crystallographic data collection and refinement statistics for several mutant structures. Metrics for highest resolution shell given in parentheses.

	I38T + 1	I38T + 2	I38T + 3	I38T + 4	E23K + 4	WT + 3	WT + 4
PDB Entry Code	**9PMP**	**9PMR**	**9PN2**	**9PN3**	**9PNL**	**9PNM**	**9PO6**
Resolution range	37.40–2.49	37.84–2.25	65.05–2.39	65.03–2.36	65.16–2.21	65.12–2.14	65.09–2.17
	(2.68–2.49)	(2.37–2.25)	(2.58–2.39)	(2.44–2.36)	(2.27–2.21)	(2.20–2.14)	(2.22–2.17)
Space group	P 62 2 2	P 62 2 2	P 62 2 2	P 62 2 2	P 62 2 2	P 62 2 2	P 62 2 2
Cell dimensions *a*,	74.8094 74.8094	75.6706 75.6706	75.112	75.357	75.196	75.194	75.161
*b, c* (Å)	118.947 90	119.472 90	75.112118.972 90	75.357121.637 90	75.196121.189 90	75.194119.641 90	75.161118.585 90
α, β, γ (°)	90,120	90,120	90,120	90,120	90,120	90,120	90,120
Unique reflections	7367 + B3	14,328	16,274	14,854	14,959	14,775	17,819
Completeness (%)	99.8 (97.9)	99.9 (99.2)	100.0 (99.8)	100.0 (100.0)	99.9 (100.0)	100.0 (100.0)	100.0 (99.7)
Mean I/sigma(I)	11.5 (2.0)	35.7 (2.1)	12 (2.0)	21 (1.8)	21 (1.0)	28 (1.4)	21 (1.8)
*R*-merge	0.012 (0.186)	0.160 (1.156)	0.101 (0.434)	0.065 (0.591)	0.067 (1.088)	0.049 (0.781)	0.053 (0.452)
*R*-measured	0.018 (0.262)	0.162 (1.171)	0.104 (0.446)	0.067 (0.607)	0.069 (1.119)	0.050 (0.803)	0.055 (0.465)
*R*-work	0.2222 (0.3076)	0.2141 (0.2693)	0.2086 (0.2817)	0.2233 (0.3368)	0.2238 (0.3344)	0.2250 (0.3350)	0.2269 (0.3001)
*R*-free	0.2659 (0.3314)	0.2540 (0.3031)	0.2675 (0.2823)	0.2792 (0.3336)	0.2678 (0.4088)	0.2741 (0.3700)	0.2721 (0.3274)
RMS(bonds)	0.005	0.005	0.002	0.008	0.004	0.004	0.009
RMS(angles)	0.7	0.77	0.44	0.89	0.62	0.62	0.98
Ramachandran favored (%)	97.09	97.11	97.7	93.18	96.57	97.16	96.02
Ramachandran outliers (%)	0	0	0	0.57	0.57	0.57	0.57
Average B-factor	76.7	66.2	60.0	65.4	68.9	72.0	56.9

**Table 3:** Thermal shift values of PA_N_ endonuclease variants with compounds **1–4** (Δ*T*_M_, C). Experiments were conducted at a concentration of 200 μM and were averaged from six independent measurements.

	WT Δ*T*_m_	I38T Δ*T*_m_	E23K Δ*T*_m_
**1**	11.91 ± 0.11	9.18 ± 0.08	5.84 ± 0.28
**2**	11.82 ± 0.13	11.16 ± 0.14	5.78 ± 0.20
**3**	10.89 ± 0.12	10.86 ± 0.12	7.21 ± 0.15
**4**	6.46 ± 0.16	4.60 ± 0.17	9.91 ± 0.30

**Table 4:** Apparent dissociation constant values (in μM) of PA_N_ endonuclease variants with compounds **1–4** (*K*_d_) and 95% CI. Experiments were conducted using thermal shift values at concentrations of 0, 25, 50, 100, 200, and 1000 μM. Measurements were performed in triplicate, and curves were generated using GraphPad Prism.

	WT *K*_d_	I38T *K*_d_	E23K *K*_d_
**1**	5.18 ± 0.93	6.84 ± 1.02	34.37 ± 9.21
**2**	4.57 ± 0.90	3.06 ± 0.45	7.18 ± 1.29
**4**	12.34 ± 4.19	10.28 ± 1.38	9.31 ± 2.86

## Table of abbreviations

CI: confidence interval
DSF: Differential Scanning Fluorometry
HOPO: hydroxypyridinone
MBP: metal-binding pharmacophore
PA_N_: RNA-dependent RNA polymerase acidic N-terminal endonuclease
WT: wild-type
